# Combined Assessment of Immune Checkpoint Regulator VISTA on Tumor-Associated Immune Cells and Platelet-to-Lymphocyte Ratio Identifies Advanced Germ Cell Tumors with Higher Risk of Unfavorable Outcomes

**DOI:** 10.3390/cancers13081750

**Published:** 2021-04-07

**Authors:** Rafał Pęksa, Michał Kunc, Marta Popęda, Michał Piątek, Michał Bieńkowski, Jolanta Żok, Anna Starzyńska, Adrian Perdyan, Marek Sowa, Renata Duchnowska, Wojciech Biernat

**Affiliations:** 1 Department of Pathomorphology, Medical University of Gdansk, 80214 Gdansk, Poland; mkunc@gumed.edu.pl (M.K.); michal.bienkowski@gmail.com (M.B.); biernat@gumed.edu.pl (W.B.); 2 Laboratory of Translational Oncology, Intercollegiate Faculty of Biotechnology, Medical University of Gdansk, 80211 Gdansk, Poland; marta.popeda@gumed.edu.pl; 3 Department of Clinical Oncology/Chemotherapy, St Barbara Regional Specialist Hospital No 5, 41200 Sosnowiec, Poland; michalpiatek@wss5.pl; 4 Department of Oncology, Regional Oncology Center in Gdansk, 80219 Gdansk, Poland; jola2019@wp.pl; 5 Department of Oral Surgery, Medical University of Gdansk, 80211 Gdansk, Poland; anna.starzynska@gumed.edu.pl; 6 Student Scientific Circle of Pathomorphology, Medical University of Gdansk, 80214 Gdansk, Poland; 532at@gumed.edu.pl; 7 Department of Urology, Medical University of Gdansk, 80214 Gdansk, Poland; marek.sowa@gumed.edu.pl; 8 Department of Oncology, Military Institute in Warsaw, 01755 Warsaw, Poland; rdtt@wp.pl

**Keywords:** testicular cancer, germ cell tumor, PD-L1, VISTA, tumor microenvironment, biomarker, prognosis, immunohistochemistry, inflammation

## Abstract

**Simple Summary:**

Testicular germ cell tumors (GCTs) are the most common malignancies in young males. The current treatment regimens are usually highly effective and curative. Nevertheless, a portion of patients presents with recurrence or succumbs due to the disease. There is an undoubtful necessity to investigate new prognostic markers to stratify the risk of such events. The current study aimed to evaluate the prognostic significance of markers of the tumor microenvironment and systemic inflammation markers in GCTs. We found that low expression of immune checkpoint proteins VISTA (V-domain Ig suppressor of T cell activation) and PD-L1 (programmed death-ligand 1) on tumor-associated immune cells and elevated inflammatory marker platelet-to-lymphocyte ratio are associated with a higher risk of events in testicular GCTs. It indicates a role of both local anti-tumor immune response and systemic inflammation in these tumors.

**Abstract:**

In the current study, we aimed to investigate whether expression of immune checkpoint proteins (V-domain Ig suppressor of T cell activation (VISTA) and programmed death-ligand 1 (PD-L1)) and markers of systemic inflammation could predict progression/relapse and death in the cohort of 180 patients with testicular germ-cell tumors (GCTs). Expression of PD-L1 and VISTA was assessed by immunohistochemistry utilizing tissue microarrays. To estimate systemic inflammation neutrophil-to-lymphocyte ratio (NLR), lymphocyte-to-monocyte ratio (LMR), and platelet-to-lymphocyte ratio (PLR) were calculated. We found high PD-L1 and VISTA expression on tumor-associated immune cells (TAICs) in 89 (49.44%) and 63 (37.22%) of GCTs, respectively, whereas tumor cells besides trophoblastic elements were almost uniformly negative. High PD-L1 was associated with seminomatous histology and lower stage. Relapses in stage I patients occurred predominantly in cases with low numbers of PD-L1 and VISTA-expressing TAICs. In stage II/III disease, the combination of low VISTA-expressing TAICs and high PLR was identified as predictor of shorter event-free survival (HR 4.10; 1.48–11.36, *p* = 0.006) and overall survival (HR 15.56, 95% CI 1.78–135.51, *p* = 0.001) independently of tumor histology and location of metastases. We demonstrated that the assessment of immune checkpoint proteins on TAICs may serve as a valuable prognostic factor in patients with high-risk testicular GCTs. Further study is warranted to explore the predictive utility of these biomarkers in GCTs.

## 1. Introduction

Testicular germ cell tumors (GCTs) are the most common type of solid malignancy in males during adolescence and young adulthood [1]. Fortunately, the majority of patients presents with stage I disease, where the risk of relapse is low and of death-negligible. To further decrease the risk of recurrence, this group may be managed by either surveillance or adjuvant chemotherapy. In nonseminomatous tumors the potential benefit from adjuvant treatment is determined based on lymphovascular invasion (LVI) [2]. On the other hand, identification of seminoma patients suitable for adjuvant carboplatin is more controversial, but rete testis invasion and tumor size are postulated as potential risk factors for occult metastatic disease [3]. Recurrent disease is almost always curable, yet the surgical or systemic treatment of relapse (cisplatin-based curative chemotherapy) carries a risk of adverse effects. Thus, more adequate selection of patients at risk of relapse may improve management of stage I disease. Patients with advanced, metastatic disease (stage IIC-III) undergo risk stratification according to International Germ Cell Consensus Classification (IGCCC), which considers tumor site (mediastinal vs. others), presence of non-pulmonary visceral metastases as well as the serum tumor markers levels: alpha-fetoprotein (AFP), βhuman chorionic gonadotropin (βhCG) and lactic dehydrogenase (LDH) [4]⁠. Albeit well-established and clinically useful, this score mostly reflects the tumor burden. Therefore, there is an undeniable need for new functional biomarkers (both prognostic and predictive) improving the current management in both early (stage I) and advanced (stage II–III) testicular cancer.

Over the last decade, the multilevel interplay between neoplasms and the immune system has gathered broad attention, with both systemic and local inflammatory response playing a significant part [5,6]. Tumor infiltrating lymphocytes (TILs) whose interactions with cancer cells are mediated by immune synapses, are an important tumor microenvironment component [7]. Binding of immune checkpoint regulators, such as V-domain Ig suppressor of T cell activation (VISTA) or programmed cell death protein 1 (PD-1) may attenuate the activity of T lymphocytes enabling immune evasion [8]. Intriguingly, the expression of VISTA and programmed death-ligand 1 (PD-L1) on TILs is associated with a favorable prognosis in some malignancies [9,10,11,12,13] and their role is probably context-dependent. The inhibition of PD-1/PD-L1 axis is effective in eliciting anti-tumor responses and improves survival in numerous malignancies, however only few clinical trials incorporated patients with GCTs. Even less is known about the prognostic and predictive role of VISTA with no reports on its expression in testicular tumors. VISTA is constitutively expressed in naïve CD4+ T cells, and normalizes innate and adaptive immune response independently of PD-L1 [14,15,16], thus, it is reasonable to compare their expression in testicular cancer. Moreover, early preclinical reports have shown enhanced antitumor T-cell responses following VISTA blockade [14].

To date, several groups investigated the role of tumor immune microenvironment in GCTs, mainly focusing on the PD-1/PD-L1 axis. It was shown that seminomas are frequently infiltrated by abundant PD-L1(+) lymphocytes and macrophages, which primarily localize in the fibrovascular septa [17]. Some studies postulated the prognostic impact of PD-L1 expression on tumor cells and immune cells in GCTs [18,19]. Other immune checkpoints previously assessed in GCTs include cytotoxic T cell antigen 4 (CTLA-4) and T cell immunoreceptor with Ig and ITIM domains (TIGIT), but their prognostic value have not been assessed yet [19,20]. Tumor immune microenvironment of GCTs was comprehensively analyzed by Siska et al. who demonstrated that advanced stage tumors are infiltrated with more regulatory T-cells and show decreased NK cell signature, while increased neutrophil signature [21]. All these findings emphasize the importance of local immune response in biology of GCTs.

Cancer influences the immune system not only at the tumor site but also globally, evoking systemic inflammatory responses. Several markers of systemic inflammation (including CRP, calcitonin, albumin, fibrinogen, etc.) are routinely monitored; however, the focus has recently been placed on a simpler alternative, easily accessible from complete blood count (CBC) results [22]. Neutrophil-to-lymphocyte ratio (NLR), lymphocyte-to-monocyte ratio (LMR), and platelet-to-lymphocyte ratio (PLR) are considered a simple measure of systemic inflammatory response and carry potentially prognostic information in various cancers [6,23,24]. Relative lymphopenia reflected by elevated NLR and PLR, or decreased LMR, may result in a lower number of TILs and attenuated anti-tumor immune response [25]. Systemic inflammation promotes tumor immune escape and facilitates invasion and metastatic spread. It was recently shown that inflammation alters the function of VISTA [16], and affects the response to PD-1/PD-L1 inhibitors [26]. Activation of platelets correlates with PD-1/PD-L1 signaling and boosts the proliferation of regulatory T cells [27]. These associations are probably reciprocal, since a decline in NLR frequently follows immune checkpoint blockade [28]. It provides a rationale for a combined evaluation of systemic inflammation markers and tumor microenvironment in cancer patients.

Additionally, a recent study by Chovanec et al. suggested that the combination of PD-L1 expression in TILs with systemic inflammatory index (SII-based on platelet, neutrophil and lymphocyte counts) may be of prognostic value in advanced GCTs [29]. Thus, it seems that insights into both systemic inflammation and the local response within the tumor microenvironment may accurately reflect tumor biology and patient’s immune condition.

In the present study, we investigated the expression and prognostic value of immune checkpoint proteins, PD-L1 and VISTA, in combination with systemic inflammatory markers in the cohort of testicular GCTs.

## 2. Materials and Methods

### 2.1. Study Group

**Figure 1:**
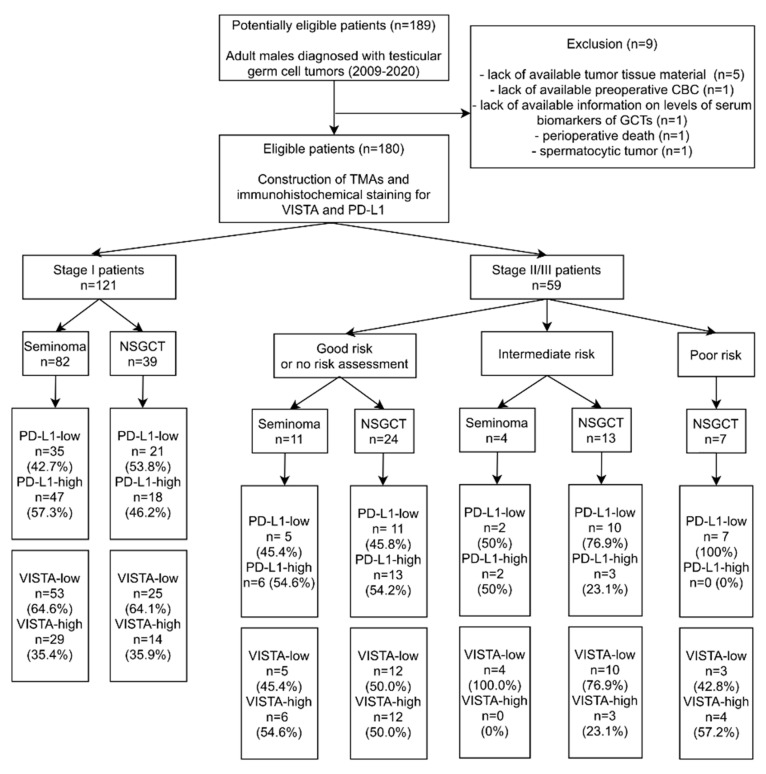
Flow chart with the patients included in the study. Abbreviations: CBC—complete blood count; GCTs—germ cell tumors; NSGCT—nonseminomatous germ cell tumor; TMAs—tissue microarrays; PD-L1—Programmed death-ligand 1; VISTA—V-domain immunoglobulin suppressor of T cell activation.

Medical records of 189 patients who had undergone orchiectomy due to GCT at the University Clinical Center in Gdańsk between January 2009 and June 2020 were retrieved using MedStream Designer, which automatically anonymizes the patients’ data. The following data were extracted: age, histological diagnosis, stage according to American Joint Committee on Cancer (AJCC) (including primary tumor-pT, status of regional lymph nodes-N, distant metastases-M, and serum biomarkers-S), metastatic sites, adjuvant chemotherapy application, CBC prior to surgery as well as the dates of diagnosis, progression, recurrence, death, and last follow-up. Subsequently, NLR, LMR, and PLR values were calculated. Nine patients were excluded from the study, leaving 180 patients in the final group (study flow-chart shown in Figure 1).

The baseline characteristics of the study group are shown in Table 1. The majority of patients had stage I disease and histology of pure seminoma. Adjuvant chemotherapy was administered in 32/82 stage I seminomas (carboplatin), and in 23/39 stage I nonseminomas (bleomycin, etoposide, and cisplatinum; BEP). One patient with stage I seminoma was treated with adjuvant radiation therapy. All patients with metastases were treated with conventional chemotherapy. Distant visceral or nodal metastases at the time of diagnosis were present in 23 patients (12.78%), whereas regional nodal metastases occurred in 55 patients (30.56%). In the whole cohort there were 11 cases of progression (6.11%) and 12 cases of recurrence (6.67%), while 8 patients (4.44%) died during the follow-up. Median follow-up time was 31.44 months (IQR: 12–58).

The study was approved by the Bioethical Committee of Medical University of Gdańsk (approval No NKBBN/485/2019).

### 2.2. Histological Examination

Histological review of all cases was performed by two pathologists (RP and MK) and the TNM staging was updated to the 8th edition of the American Joint Committee on Cancer Staging Manual when necessary [30].

Tissue microarrays (TMAs) were constructed with Manual Tissue Arrayer MTA-1 (Beecher Instruments, Inc., Sun Praire, WI, USA) using 1.5 mm core needles. Representative tumor areas from the periphery and central portion of the tumor sample were selected (in mixed tumors each component was sampled). In rare cases with poorly-fixed under-processed central part of the tissue block, only the peripheral area was sampled. The median number of cores per patient equaled 3 (range: 2–10). All cores were examined for the presence of tumoral/peritumoral lymphocytes and macrophages, further termed tumor-associated immune cells (TAICs). Tumor necrosis was assessed in all cases, since it may possibly affect the systemic inflammatory response.

All TMAs were stained with the antibodies against PD-L1 (clone 22C3, 1:50 dilution, DAKO) and VISTA (clone D5L5T, 1:300 dilution, Cell Signaling) with the appropriate positive (nonneoplastic tonsil and placenta cores) and negative (nonneoplastic liver) controls incorporated into TMAs. The stainings were independently evaluated by two pathologists (RP and MK) for tumor cells and TAICs separately; for discrepant cases, a consensus score was settled. The positive staining was defined as a distinct, complete membranous pattern in tumor cells, while either membranous or cytoplasmic in tumor-associated immune cells. For PD-L1, the stainings were assessed by a weighted histoscore as described by Chovanec et al. [18]. The final score was obtained by calculation of mean score from several cores and graded as low (0–40) or high (41–300). Due to negligible differences in intensity of VISTA staining only the percentage of positive cells was evaluated, and then subclassified into low (0–50%), or high (50–100%) expression status. In cases with discrepant staining results between cores, the dominant pattern was noted.

### 2.3. Statistical Analysis

Receiver operating characteristic (ROC) curves were plotted for NLR, LMR, and PLR vs. any event (relapse, progression, or death). Values discriminating stage I and stage II/III cases were determined analogously. The optimal cut-off values were selected based on the maximal Youden’s index and used for dichotomization.

The associations between PD-L1, VISTA, CBC-derived factors and clinicopathological characteristics (age, stage, and histology) were assessed by U-Mann Whitney test for continuous variables, and Chi square test or Fisher’s exact test for categorical variables.

Progression in advanced tumors was defined as either enlargement of the residual mass after initial response or development of new metastases during treatment. Relapse was defined as metastatic disease developed after remission. Event-free survival (EFS) was defined as the time from the diagnosis until the relapse or progression (in stage II-III tumors). Cancer-specific survival (CSS) was defined as the time from the diagnosis until death due to testicular cancer.

Logistic regression and Cox proportional hazard analysis were performed to estimate the odds ratios or hazard ratios (OR or HR, respectively) and 95% confidence intervals (CIs). Backward selection was employed to create a multivariable model predicting event and to eliminate nonsignificant variables at *p* < 0.05. Differences in EFS between groups were assessed using log-rank test and visualized with Kaplan–Meier curves. Statistical analysis was performed with Statistica 13.3 software (TIBCO Software Inc., Palo Alto, CA, USA) and R statistical environment [31]. Boxplots were plotted using the “ggplot2” package [32]. Kaplan–Meier curves were plotted using the “survminer” and “ggsci” packages [33,34].

## 3. Results

### 3.1. Expression of PD-L1 and VISTA in Testicular Germ Cell Tumors

**Figure 2:**
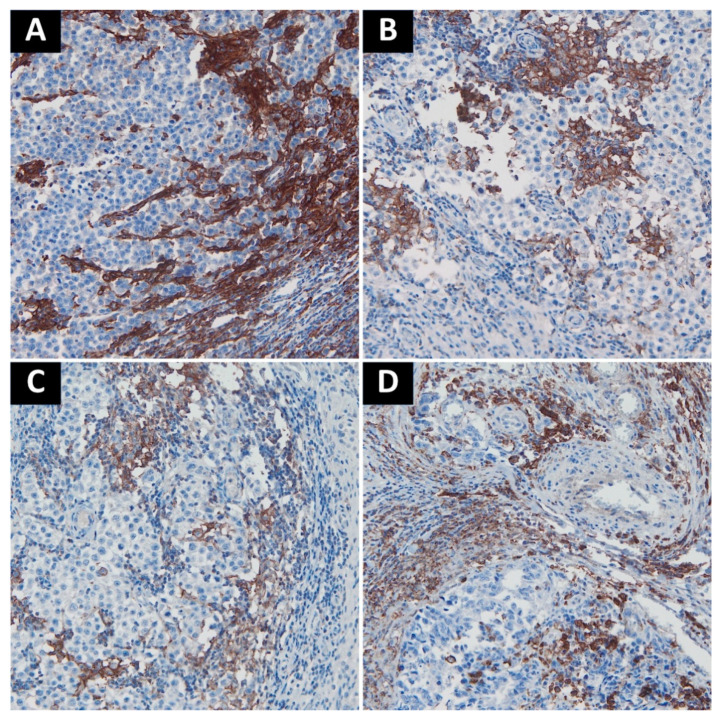
Representative examples of PD-L1 staining. (**A**). Intense staining in TAICs along the interface between fibrovascular septa and tumor cells; (**B**). Moderately positive to negative TAICs in seminoma; (**C**). Weakly positive to negative TAICs in seminoma; (**D**). Embryonal carcinoma with moderate PD-L1 staining in TAICs. Abbreviations: PD-L1: programmed death-ligand 1; TAICs: tumor-associated immune cells.

**Figure 3:**
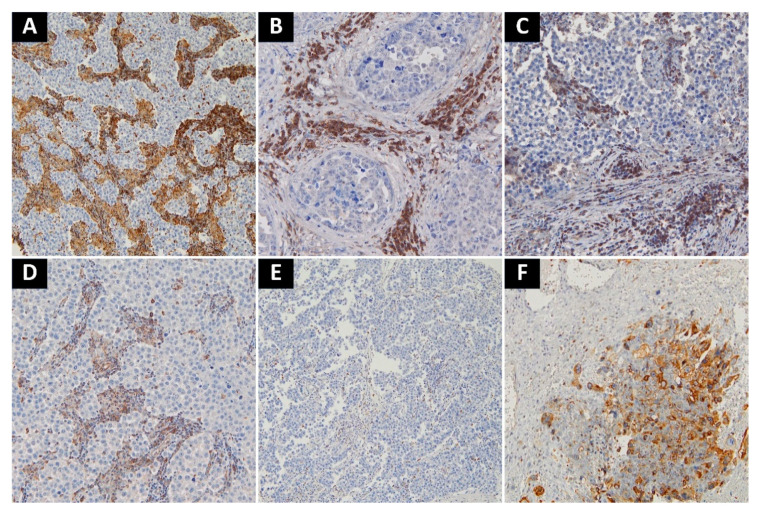
Representative examples of VISTA-staining. (**A**) Seminoma with intense VISTA staining in TAICs in fibrovascular septa; (**B**) Embryonal carcinoma with intense staining in TAICs; (**C**) Scattered VISTA-positive TAICs in stroma surrounding embryonal carcinoma; (**D**) Moderate amount of VISTA-positive TAICs in fibrovascular septa of seminoma; (**E**) Single VISTA-positive TAICs in seminoma; (**F**) VISTA-positive. choriocarcinoma cells in mixed malignant germ cell tumor. Abbreviations: VISTA: V-domain Ig suppressor of T cell activation; TAICs: tumor-associated immune cells.

High expression of both VISTA and PD-L1 was noted in the choriocarcinoma component. Otherwise, tumor cells were negative for both markers, except for 3 embryonal carcinoma cases with a focal PD-L1 staining. Tumor-associated immune cells with high VISTA expression were observed in 63 cases (37.22%), while those with high PD-L1 expression in 89 cases (49.44%). A complete lack of PD-L1 expression was noted in seven seminomas (7.2% of seminomas), and three cases of MGCT (3.6% of nonseminomas). Only two cases of seminoma showed complete absence of VISTA+ cells (2.1% of seminomas). Pure teratomas and areas of teratoma in mixed tumors were uniformly characterized by low number of TAICs and very weak or absent expression of immune checkpoints (PD-L1 histoscore < 5, percentage of VISTA+ cells < 5%). As previously described, in seminomas PD-L1-expressing TAICs were distributed mainly along the tumor interface and within the fibrovascular septa [17] (Figure 2A–C)⁠, while VISTA-positive TAICs were mainly located within septa with a less prominent interface enhancement (Figure 3A,D,E). In nonseminomatous tumors, VISTA-expressing cells frequently formed small clusters within tumor parenchyma or in peritumoral borders of fibrovascular septa (Figure 3B,C), while the distribution of PD-L1 positive TAICs was more heterogeneous and patchy (Figure 2D). Non-neoplastic Leydig cells and endothelia showed VISTA expression regardless of tumor histology.

High expression of PD-L1 was more prevalent in pure seminomas than in other types of germ cell tumors. Tumors disseminated to lymph nodes or distant sites displayed lower expression of PD-L1 on TAICs. On the other hand, VISTA expression was not significantly associated with any analyzed clinicopathological variable except PD-L1. Associations between PD-L1 and selected clinicopathological variables are shown in Table 2.

Stage I and good risk stage II and III GCTs tended to have a higher frequency of PD-L1-high tumors than the advanced intermediate/poor risk cases. This trend was observed for both seminomas and nonseminomatous GCTs (Figure 1).

### 3.2. Markers of Systemic Inflammation

**Figure 4:**
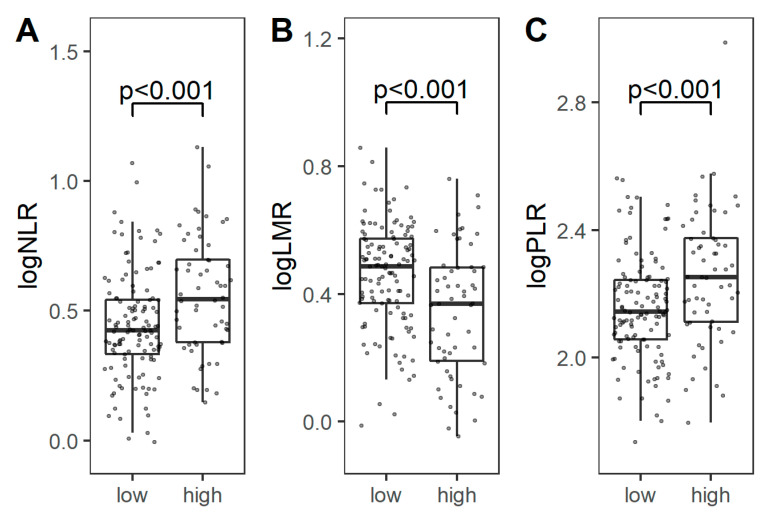
Comparison of logarithmic NLR (**A**), LMR (**B**), and PLR (**C**) between low-stage (I) and high-stage (II/III) tumors. Abbreviations: NLR: neutrophil-to-lymphocyte ratio; LMR: lymphocyte-to-monocyte ratio; PLR: platelet-to-lymphocyte ration.

**Figure 5:**
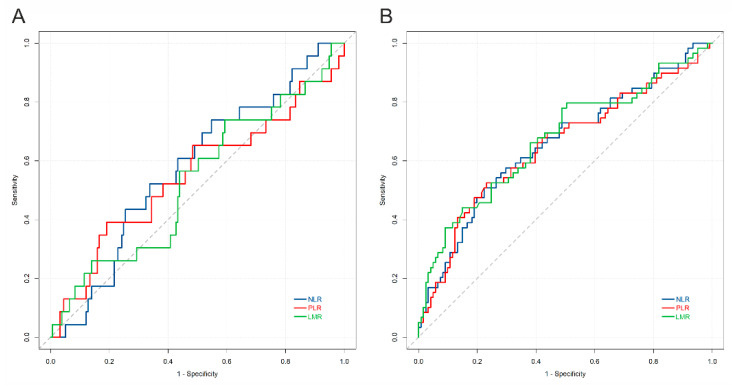
Receiver operating curves for NLR, LMR, PLR, versus (**A**) event (relapse, progression, or death), and (**B**) high stage. The AUC for the prediction of event was: NLR = 0.57; PLR = 0.54; LMR = 0.53; the AUC for the prediction of stage was: NLR = 0.65; PLR = 0.65; LMR = 0.67. Abbreviations: NLR: neutrophil-to-lymphocyte ratio; LMR: lymphocyte-to-monocyte ratio; PLR: platelet-to-lymphocyte ration.

The levels of NLR, LMR, and PLR were not dependent on tumor histology and expression of immune checkpoints on TAICs. However, higher NLR or PLR and lower LMR were associated with the presence of nodal or distant metastases, and higher stage (Figure 4). The presence of tumor necrosis was correlated with higher NLR and lower LMR (*p* = 0.004 and 0.001, respectively; U-Mann–Whitney). The calculated cut-off values for NLR, LMR and PLR to predict events were 3.95, 3.09, and 212, respectively (Figure 5A). All three markers could predict stage, as demonstrated by ROC analysis, and the estimated cut-off values for NLR, LMR, and PLR were 3.56, 3.08, and 175, respectively (Figure 5B).

### 3.3. Survival Analysis

#### 3.3.1. Survival Analysis in the Whole Cohort

**Figure 6:**
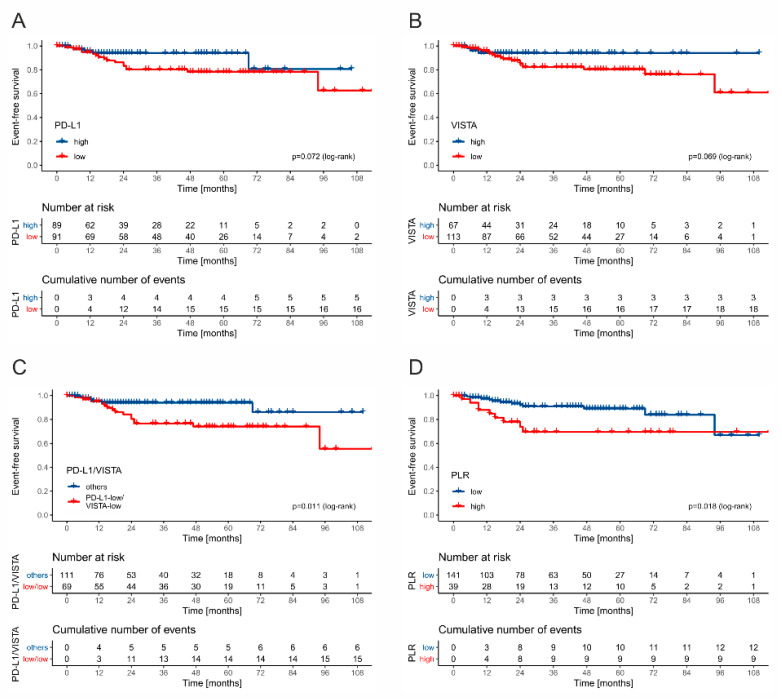
The Kaplan–Meier curves for event-free survival stratified by PD-L1 (**A**), VISTA (**B**), combined PD-L1 and VISTA (**C**), and PLR (**D**).

The structure of events in the whole cohort according to the stage including ratio of immune checkpoint receptor-high to -low cases is shown in Table 3. The five-year EFS rate was lower in tumors displaying low PD-L1 expression on TAICs, when compared to tumors infiltrated with abundant PD-L1(+) cells (78% vs. 94%, *p* = 0.072) (Figure 6A). Similarly, high VISTA expression was associated with a more favorable 5-year EFS rate, when compared to VISTA-low tumors (94% vs. 80%, *p* = 0.069) (Figure 6B). Tumors with both low PD-L1 and VISTA expression on TAICs showed worse five-year EFS than other tumors (74% vs. 94%, *p* = 0.011). Taking into consideration the preoperative systemic inflammatory markers, the five-year EFS rate was significantly lower in the PLR-high (89%) than in the PLR-low (69%) group (*p* = 0.018). Neither NLR nor LMR were significantly associated with survival rate.

#### 3.3.2. Assessment of Risk of Relapse in Stage I Patients

**Figure 7:**
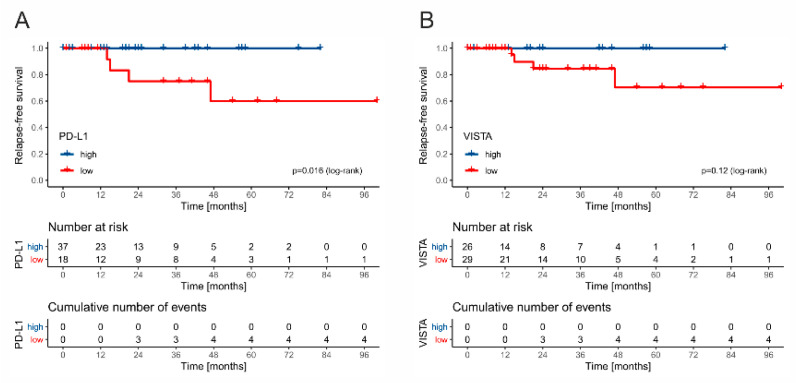
The Kaplan–Meier curves for relapse-free survival in stage I patients treated with adjuvant chemotherapy stratified by PD-L1 (**A**) and VISTA (**B**).

Due to different management and frequency of events between stage I and stage II/III patients, we decided to separate these groups in survival analysis. Among all stage I GCT cases, only two patients died during the follow-up (2/121, and 1.65%) but it was not directly related to testicular cancer. There were six cases of relapse (4.95% in total; 4/82; 4.87% of seminomas; 2/39, 5.12% of nonseminomas). Patient management (surveillance vs. adjuvant chemotherapy) was not associated with recurrence rate, but this analysis is underpowered. The low number of observed relapses in this cohort may possibly result from the transfer of stage I patients to smaller centers and loss to follow-up before the onset of late recurrences. All six relapsing cases were designated as VISTA-low, and five as PD-L1-low. In the group of patients receiving adjuvant chemotherapy, low expression of PD-L1 was significantly associated with RFS (*p* = 0.016, log-rank) (Figure 7A), and a similar trend was observed for VISTA (*p* = 0.12, log-rank) (Figure 7B), whereas the infiltration of rete testis, tumor size, LVI, tumor extent (pT feature), and markers of systemic inflammation did not affect the risk.

#### 3.3.3. Survival Analysis in Stage II/III Disease

**Figure 8:**
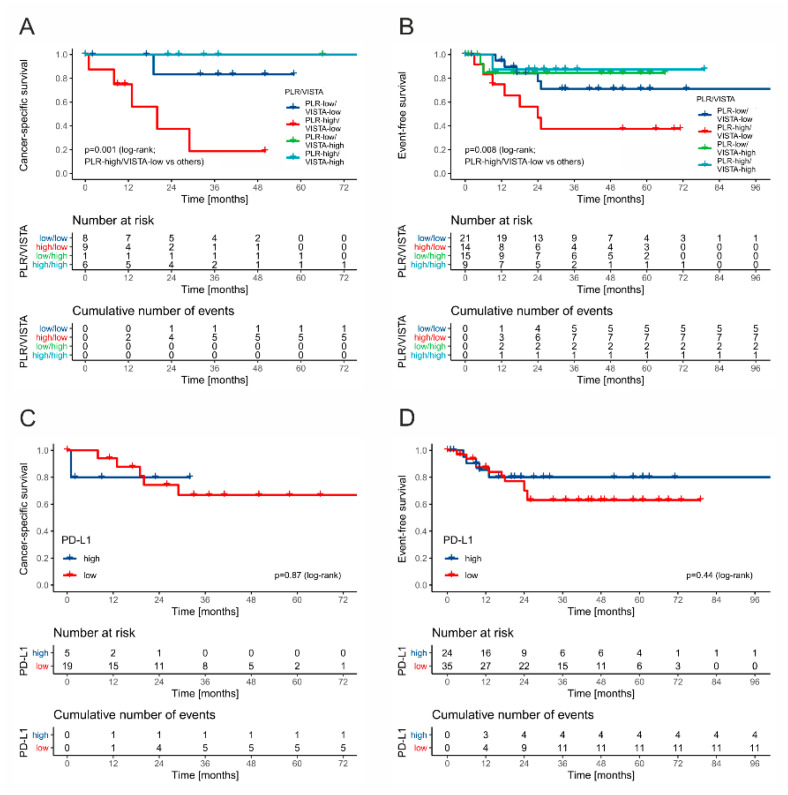
The Kaplan–Meier curves for event-free survival and cancer-specific survival in stage II/III patients stratified by VISTA and PLR ((**A**) and (**B**), respectively) and PD-L1 ((**C**) and (**D**), respectively).

Patients with stage II/III disease presented with 11 cases of progression (11/18.64%), six relapses (11.1%), and six cancer-specific deaths (11.1%). All deaths occurred in patients with intermediate (5/17, 29.41%) or poor (1/7, 14.28%) IGCCCG group. Thus, the CSS analysis was restricted to this subgroup, whereas EFS was analyzed for all stage II/III patients. Low VISTA expression was associated with a higher risk of events and death (*p* = 0.1031 and *p* = 0.054, respectively). Next, the combination of VISTA and PLR stratifies the patients in terms of EFS and CSS (poorest outcomes in VISTA-low/PLR-high cases, while best in VISTA-high cases; Figure 8A,B). PD-L1 expression and markers of systemic inflammation alone had no impact on EFS and OS (Figure 8C,D). The combined low expression of VISTA on TAICs and high PLR was identified as an independent predictor of relapse/progression (HR 4.10; 1.48–11.36, *p* = 0.006) and death (HR 15.56, 95% CI 1.78–135.51, *p* = 0.001) in multivariate Cox regression analysis.

#### 3.3.4. Comparison of Factors Influencing Survival in Seminomas and Nonseminomas

Due to the different biology and treatment of seminomas and nonseminomatous GCTs, we investigated the prognostic value of immune checkpoints and systemic inflammatory markers in histologic subgroups using logistic regression. In seminoma group, PD-L1 and serum markers were associated with the risk of relapse or progression (Table 4). In contrast, the prognosis of nonseminomas was affected by the baseline presence of metastases and combined status of VISTA and PLR (Table 5).

## 4. Discussion

Our study aimed to investigate the associations between immune checkpoint proteins, PD-L1 and VISTA, systemic inflammatory markers and the prognosis in a cohort of testicular GCTs. We demonstrated that low expression of VISTA combined with high levels of PLR is associated with the risk of dismal outcomes in stage II/III GCTs. The results support the hypothesis that both local tumor immune microenvironment and systemic inflammatory response influence the clinical behavior of GCTs. Moreover, an analysis restricted to stage I tumors treated with adjuvant chemotherapy revealed that low expression of PD-L1 may predict an increased risk of relapse. Nevertheless, due to the low number of events in stage I patients, any conclusions have to be drawn with caution.

Testis is one of the immune privileged organs in the human body, where haploid germ cells expressing novel autoantigens are protected from immunological elimination by the blood–testis barrier formed by Sertoli cells [35]. Other cells inhabiting testis regulate local immune responses by the production of immunosuppressive agents. Leydig cells directly interact with immune cells, and their secretion of immunosuppressive substances is regarded as one of the autoimmune prevention mechanisms [36]. In the current study, we demonstrated the consistent expression of VISTA in Leydig cells, which was not previously described. Loss of these immunosuppressive mechanisms results in autoimmune orchitis. Importantly, it could be an adverse effect of anti-PD1 therapy, emphasizing the role of immune checkpoints in maintaining testicular immune homeostasis [37]. On the contrary, the lack of adequate immune surveillance may participate in the development of GCTs [38]. To achieve an effective anti-tumor response, testis probably needs to abnegate its immune privilege, similarly as it is during microbial infections. It is possible that in rare cases it may even lead to regression of the tumor, explaining the phenomenon of so called “burned-out” testicular cancers [39].

The interactions between the inflammatory infiltrates and GCTs have been investigated since Marshall et al. described aggregates of lymphocytes and granulomas in seminomas and dysgerminomas in 1964 [40]. Hadrup et al. demonstrated the presence of cytotoxic and clonally expanded melanoma-associated antigen 3 (Mage-3) specific T cells among TILs in seminoma, which suggests that the inflammatory infiltrate serves to maintain the immunological control of the tumor [41]. CD8+ and CD4+ T-cells responsive against MAGE-A family antigens were also detected in the peripheral blood of seminoma patients, supporting the systemic surveillance of testicular cancer [42]⁠. On the other hand, the reports on PD-1/PD-L1 axis in testicular GCTs are relatively sparse. A study on murine models showed a constitutive PD-L1 expression in spermatocytes and spermatids in seminiferous tubules, contributing to testicular immune privilege [43]. Nevertheless, we and other groups observed no PD-L1 expression in normal testicular tissue, which may reflect the differences in testicular immunity between human and mouse [18,19]. In 2016, Cierna et al. showed that none of the GCTs in their cohort exhibited PD-1 expression, whereas a high PD-L1 expression on tumor cells (noted in about 20% of cases) was associated with poor prognostic factors and worse survival [18]. Their subsequent study on TILs within GCTs demonstrated a favorable prognosis of tumors with PD-L1-positive TILs, especially in cases with PD-L1-negative tumor cells [44]. In contrast, we rarely noted PD-L1 expression on tumor cells, which may be due to the fact that Cierna and colleagues used a different antibody clone, i.e., Abcam [EPR1161(2)], which is not a clinically validated one. Furthermore, distinguishing the expression of PD-L1 between tumor cells and TILs is not always clear, especially in tumor nest borders and fibrovascular septa. This difficulty was overcome by Sadigh et al. by means of dual immunohistochemical stains for OCT3/4 and PD-L1 (clone 22c3) to reveal no true PD-L1 positivity in tumor cells [45].

Moreover, we observed a trend towards lower expression of both immune checkpoint proteins, especially PD-L1, in metastatic high-risk patients compared to stage I and low risk cases. It may indicate that advanced GCTs constitutionally express lower levels of immune checkpoint receptors or modulate their expression during progression. Similar observations were made by Siska and colleagues [21].

The favorable prognosis in GCTs with high PD-L1 or VISTA expression on TAICs is not easily explained. We hypothesize that tumor microenvironment of GCTs is unique and characterized by the lack of PD-1/PD-L1 expression on tumor cells with the abundance of TAICs expressing immune checkpoint proteins, especially in early stage patients. In this scenario the presence of PD-L1 or VISTA expression on TAICs may reflect pre-existing immunity, as described in head and neck squamous cell carcinomas [10]. High expression of VISTA on immune cells is associated with favorable prognosis and abundant TILs in breast and lung cancers [46,47]. Likewise, PD-L1 expression on macrophages correlates with an activated immune microenvironment and prolonged survival in hepatocellular carcinoma [48], and, importantly, in primary testicular lymphoma [49]. Alternatively, the expression of immune checkpoint proteins may reduce the proinflammatory environment, thus, inhibiting the so-called tumor-promoting inflammation [5,38].

In pediatric patients with extracranial GCTs, high CD3+ T-cell infiltration, both in tumor nests and septa, indicated a favorable outcome [50]. Interestingly, PD-L1-positive TILs were reported only in single cases (one yolk sac tumor and three embryonal carcinomas). It may be related to a less immunogenic potential of pediatric than adult GCTs and lack of seminomas in this age group, yet again, the use of a different antibody clone (RBT-PDL1, BioSB, Santa Barbara, CA, USA.), precludes any clear conclusions. The summary of the studies investigating the expression of PD-L1 in GCTs is presented in Table 6 [17,18,19,21,44,45,50,51].

To the best of our knowledge, this is the first study to determine the VISTA expression in GCTs, a recently described negative checkpoint regulator capable of T-cell inactivation in parallel to the PD-1/PD-L1 axis [52]. Similarly to PD-L1, expression of VISTA was mainly noted in TAICs, but not on tumor cells. Additionally, we observed a strong VISTA expression in the choriocarcinoma components, which is in line with the report by Zong et al. [53] on the wide overexpression of VISTA and PD-L1 in gestational trophoblastic neoplasms.

Moreover, the list of immune-checkpoint proteins expressed in GCTs is expanding, recently with TIGIT and CTLA-4 [19,20]⁠. Unfortunately, recent findings suggest that immune-checkpoint inhibitors (durvalumab plus tremelimumab or pembrolizumab) are mainly ineffective in patients with advanced GCTs [54,55]. Nevertheless, Zschäbitz et al. reported a long-term response to anti-PD-1 therapy only in two out of seven patients. Both responsive cases displayed a strong PD-L1 expression, suggesting that PD-L1-expressing tumors have higher likelihood of gaining benefit from immunotherapy [56]. All studies to date mainly enrolled patients with nonseminomatous GCTs irrespective of their PD-L1 status. We hypothesize that PD-L1 expression has more pronounced effects on the biology of seminomas than nonseminomas. Moreover, advanced or relapsing tumors may be characterized by a general low level of PD-L1. Alternatively, it is possible that refractory nonseminomas could be treated with inhibitors of other immune checkpoint receptors, including VISTA or dual inhibitors of both PD-L1 and VISTA, which are currently developed [57]. As we have shown the outcomes of GCTs are influenced by systemic inflammation in advanced stage patients. Recent studies on non-small cell lung carcinoma indicate that NLR values influence the prognostic significance of PD-L1 expression on tumor cells, and higher NLR correlated with a worse response to pembrolizumab [58,59]. This factor might have influenced the outcomes in testicular cancer treated with anti-PD-1/PD-L1 drugs in a similar manner. Finally, GCTs possess a unique immune microenvironment, as they lack PD-1/PD-L1 expression on tumor cells, and frequently display high expression of immune checkpoint proteins on TAICs. Hence, further translational studies should investigate if inhibition of immune checkpoints is reasonable in refractory GCTs.

Multiple studies have evaluated CBC-derived markers in various diseases, with the general trend indicating poorer outcomes in cancer patients with high NLR, high PLR, and low LMR [6]. Increased neutrophil, platelet and monocyte levels may result from a systemic inflammatory reaction, facilitating cancer progression. Two small studies showed that patients with GCTs have higher NLR and PLR values than healthy controls [60,61]⁠. Recently, high preoperative NLR was identified as a stage predictor and poor prognostic factor for overall survival by two independent groups [62,63]. Our study confirms the stage-predicting value of systemic inflammatory markers (Figure 4 and Figure 5B). Fankhauser et al. demonstrated that high levels of leukocytes, neutrophils, NLR, and SII in metastatic GCTs patients before the first-line chemotherapy indicate poorer outcomes [64]. Moreover, it was shown that pretreatment NLR and Prognostic Nutritional Index may be independent risk factors for development of bleomycin pulmonary toxicity in patients with GCTs [65]. According to Chovanec et al., PD-L1 expression on TILs and SII identify three distinct prognostic subgroups of GCTs, with PD-L1-high and SII-low patients having excellent outcomes [29]⁠. We failed to replicate these results, however we noted a trend towards worse survival in patients with PD-L1-low GCTs. We observed a similar association for VISTA expression on TAICs and PLR. These discrepancies may be due to underrepresentation of advanced seminomas in our cohort, since our data demonstrate that the expression of PD-L1 has higher prognostic significance in seminomatous GCTs. We postulate that simultaneous evaluation of tumor immune microenvironment and systemic inflammatory response could be a novel tool to identify patients at increased risk.

The relation between high preoperative PLR and wore EFS and OS has not been reported before in testicular cancer. Nevertheless, several meta-analyses reported on inferior survival in patients with various advanced solid malignancies [23,24]. This association may result from platelets protecting tumor cells from NK-cell-dependent lysis, or the secretion of growth factors [66,67]. These results suggest that both systemic and local inflammatory processes affect the clinical course of GCTs.

There are three main limitations of our study. First of all, our cohort consists of relatively low number of intermediate and poor risk stage II/III patients. Moreover, we noted a low number of events in stage I patients, which may be caused by the transfer of these patients to smaller centers and loss to follow-up before the onset of late relapses. Further, in our study many stage I patients received adjuvant chemotherapy. It should also be noted that the patients included in our study have been diagnosed from 2009 to 2020, this is a period of 11 years. Surveillance strategy in our cohort has grown as the preferred treatment after orchiectomy from 32.1% in 2004 to 81.2% in 2015 [68]. Finally, our study is based on expression assessment in TMAs, which hampers comprehensive evaluation of immune microenvironment in the whole tumor.

## 5. Conclusions

To conclude, in this retrospective study, we demonstrate the potential usefulness of combined assessment of immune checkpoint regulator VISTA and PLR in metastatic intermediate/poor risk GCTs. These biomarkers may be used to stratify the risk of progression or death and to identify the patients requiring a more aggressive therapeutic approach. The prognostic impact of PD-L1 is especially prominent in seminomas, indicating the crucial role of immune surveillance in the biology of these tumors. Evaluation of PD-L1 may be implemented to predict the risk of relapse in stage I patients treated with adjuvant therapy. However, larger, optimally prospective, studies should be performed to elucidate the prognostic role of immune checkpoint receptors and systemic inflammatory markers in GCTs. While PD-1/PD-L1 inhibitors seem to be ineffective in refractory GCTs, it is possible that interference with other immune checkpoint receptors expressed in tumor microenvironment may be potentially beneficial for these patients.

## Figures and Tables

**Table 1:** Basic characteristics of the study group. Abbreviations: EC—embryonal carcinoma; MGCT—mixed germ cell tumor; BEP—Bleomycin, etoposide and cisplatinum; PD-L1—Programmed death-ligand 1; VISTA—V-domain immunoglobulin suppressor of T cell activation; IGCCCG—International Germ Cell Cancer Collaborative Group.

Feature		
Mean Age (Max–Min) (Years)	32.89 (17–66)	
Diagnosis (*n*, %)	Seminoma	97 (53.9)
	Nonseminoma	83 (46.1)
	EC	15 (8.3)
	Teratoma	2 (1.1)
	MGCT	66 (36.7)
T (*n*, %)	1	69 (38.3)
	2	102 (56.7)
	3	8 (4.4)
	4	1 (0.5)
*n* (*n*, %)	0	125 (69.4)
	1	8 (4.4)
	2	22 (12.2)
	3	25 (13.9)
M (*n*, %)	0	157 (87.2)
	1a	19 (10.5)
	1b	4 (22.2)
S (*n*, %)	0	81 (45.0)
	1	63 (35.0)
	2	28 (15.5)
	3	8 (4.4)
Stage according to AJCC (*n*, %)	I	121 (67.2)
	II	29 (16.1)
	III	30 (16.7)
IGCCCG risk groups (stage IIC–III patients only)	Good	35 (84.4)
	Intermediate	17 (11.7)
	Poor	7 (38.8)
Adjuvant chemotherapy (stage I patients) (*n*, %)	No	66/121 (54.5)
	BEP in NSCGTs	23/39 (59.0)
	Carboplatin in seminoma	32/82 (39.0)
Event (*n*, %)	Progression	12 (6.7)
	Relapse	11 (6.1)
	Death	8 (4.4)

**Table 2:** Associations between PD-L1 expression on TAICs and dichotomized clinicopathological variables. *—statistically significant. Abbreviations: PD-L1—Programmed death-ligand 1; VISTA—V-domain immunoglobulin suppressor of T cell activation; LVI—lymphovascular invasion; TAICs—tumor-associated immune cells.

	PD-L1 Low	PD-L1 High	*p*
VISTA low	69 (38.33)	44 (24.44)	<0.001 *
VISTA high	22 (12.22)	45 (25.00)	
Pure seminoma	42 (23.33)	55 (30.56)	0.035 *
Other histologies	49 (27.22)	34 (18.89)	
LVI-1	50 (27.78)	52 (28.89)	0.637
LVI-0	41 (22.78)	37 (20.56)	
T1	35 (19.44)	33 (18.33)	0.848
T2–4	56 (31.11)	56 (31.11)	
N0	57 (31.67)	68 (37.78)	0.045 *
N1–3	34 (18.89)	21 (11.67)	
M0	74 (41.11)	83 (46.11)	0.016 *
M1	17 (9.44)	6 (3.33)	
S0–1	67 (37.22)	77 (42.78)	0.031 *
S2–3	24 (13.33)	12 (6.67)	
Stage I	56 (31.11)	65 (36.11)	0.100
Stage II–III	35 (19.44)	24 (13.33)	

**Table 3:** Frequency of events according to stage and histology. * death unrelated to testicular cancer. Abbreviations: PD-L1—Programmed death-ligand 1; VISTA—V-domain immunoglobulin suppressor of T cell activation; *n*/a—not applicable; NSGCTs—nonseminomatous germ cell tumors.

Stage	IGCCCG Risk Group *n* (%)	Relapse/ Progression *n* (%)	Death *n* (%)	Percentage of PD-L1-High Cases	Percentage of VISTA-High Cases
Seminoma					
I (*n* = 82)	N/a	4 (4.9)	2 (2.4) *	51.31%	35.36%
II/III (*n* = 15)	Good or no need for risk assessment (*n* = 11)	2 (13.3)	0 (0)	54.54%	54.54%
	Intermediate (*n* = 4)	1 (25.0)	2 (50.0)	50.00%	0.0%
NSGCTs					
I (*n* = 39)	N/a	2 (5.1)	0 (0)	46.15%	35.89%
II/III (*n* = 44)	Good or no need for risk assessment (*n* = 24)	4 (16.7)	0 (0)	54.16%	50.00%
	Intermediate (*n* = 13)	6 (46.1)	3 (23.1)	30.00%	30.00%
	Poor (*n* = 7)	2 (28.6)	1 (14.3)	0.0%	57.14%

**Table 4:** Univariate and multivariate logistic regression analysis of factors predicting event (progression or relapse) in nonseminomatous testicular germ cell tumors. *—statistically significant; ^!^—not included in multivariate analysis. Abbreviations: OR—odds ratio; PD-L1—Programmed death-ligand 1; VISTA—V-domain immunoglobulin suppressor of T cell activation; L—low expression; H—high expression; LVI—lymphovascular invasion; NLR—neutrophil-to-lymphocyte ratio; PLR—platelet-to-lymphocyte ratio; LMR—lymphocyte-to-monocyte ratio.

	Univariate		Multivariate	
Characteristics	OR (95% CI)	*p*	OR (95% CI)	*p*
Stage	3.70 (1.63–8.38)	0.002 *^,!^		
pT (1 vs. 2–4)	0.63 (0.19–2.15)	0.465		
*n* (1–3 vs. 0)	3.25 (0.93–11.38)	**0.065**		
M (1 vs. 0)	6.67 (1.89–23.51)	0.003 *	5.27 (1.30–21.38)	0.020 *
S (2–3 vs. 0–1)	3.60 (1.09–11.87)	0.035 *		
LVI	2.45 (0.76–7.89)	0.133		
PD-L1 (L vs. H)	1.92 (0.54–6.73)	0.306		
VISTA (L vs. H)	4.89 (1.02–23.53)	0.047 *		
PD-L1/VISTA (LL vs. others)	3.89 (1.11–13.66)	0.034 *		
NLR (L vs. H)	3.26 (1.00–10.62)	0.049 *		
PLR (L vs. H)	3.78 (1.15–12.38)	0.028 *		
LMR (L vs. H)	1.51 (0.43–5.31)	0.520		
PD-L1/PLR (L/H vs. others)	5.90 (1.70–20.46)	<0.001 *		
VISTA/PLR (L/H vs. others)	10.16 (2.80–36.92)	<0.001 *	8.49 (2.16–33.30)	0.002 *

**Table 5:** Univariate and multivariate logistic regression analysis of factors predicting event (progression or relapse) in seminomas. *—statistically significant; **—due to distribution of events analysis was precluded; ^!^—not included in multivariate analysis. Abbreviations: OR—odds ratio; PD-L1—Programmed death-ligand 1; VISTA—V-domain immunoglobulin suppressor of T cell activation; L—low expression; H—high expression; LVI—lymphovascular invasion; NLR—neutrophil-to-lymphocyte ratio; PLR—platelet-to-lymphocyte ratio; LMR—lymphocyte-to-monocyte ratio.

	Univariate		Multivariate	
Characteristics	OR (95% CI)	*p*	OR (95% CI)	*p*
Stage	2.59 (1.03–6.51)	0.042 *^,!^		
pT (1 vs. 2–4)	1.16 (0.24–5.51)	**0.845**		
*n* (1–3 vs. 0)	4.87 (0.96–24.53)	**0.055**		
M (1 vs. 0)	6.8 (1.04–44.19)	0.045 *		
S (2–3 vs. 0–1)	10.67 (2.08–54.71)	0.004 *	**15.05 (2.38–94.79)**	0.004 *
Infiltration of rete testis	1.97 (0.21–18.81)	0.553		
Tumor size > 4 cm	1.27 (0.27–6.02)	0.758		
PD-L1 (L vs. H)	9.00 (1.03–77.93)	0.046 *	**12.70 (1.24–129.52)**	0.032 *
VISTA (L vs. H)	3.47 (0.40–30.12)	0.258		
PD-L1/VISTA (LL vs. others)	5.83 (1.06–31.95)	0.042 *		
NLR (L vs. H)	0.62 (0.07–5.49)	0.670		
PLR (L vs. H)	1.08 (0.12–9.80)	0.943		
LMR (L vs. H)	2.49 (0.47–13.08)	0.279		
PD-L1/PLR (L/H vs. others)	1.89 (0.20–17.64)	0.575		
VISTA/PLR (L/H vs. others)	N/A **			

**Table 6:** The summary of studies investigating expression of PD-L1 in germ cell tumors. Abbreviations: FIHC—fluorescence immunohistochemistry; ChC -choriocarcinoma; EC—embryonal carcinoma; YST—yolk sac tumor; TILs—tumor infiltrating lymphocytes; TAMs—tumor associated macrophages; ICs—immune cells; GCTs—germ cell tumors; PFS—progression-free survival; RFS—relapse-free survival.

Author/Year	Fankhauser (2015) [17]	Cierna (2016) [18]	Chovanec (2017) [44]	Lobo (2019) [19]	Siska (2017) [21]	Sadigh (2020) [45]	Boldrini (2019) [50]	Jennewein (2018) [51]	Pęksa (2020) Current study
Antibody	E1L3N (monoclonal rabbit antibody; dilution 1:1000)	EPR1161(2) (monoclonal rabbit antibody; dilution 1:100)	EPR1161(2) (monoclonal rabbit antibody; dilution 1:100	22C3 (monoclonal mouse antibody; dilution 1:100)	E1L3N (monoclonal rabbit antibody; multiplexed FIHC)	E1J2J (monoclonal rabbit antibody; dilution 1:2000)	RBT-PDL1 (monoclonal rabbit antibody)	E1L3N (monoclonal rabbit antibody)	22C3 (monoclonal rabbit antibody; dilution 1:50)
Evaluation of staining/criteria for postivity	Percentage of cells. Threshold: 5% positive cells	Multiplicative quickscore (0–9—low; 10–18—high)	Weighted histoscore (0–150—low; 160–300—high)	Presence or absence of any positive cells	Automated analysis; calculation of PD-1/PD-L1 interaction score	H-score	Density of positive cells	A multi-score of staining frequency and intensity	Weighted histoscore (0–40—low; 41–300—high)
Number of cases	329	140	240	265	35	77	49 (pediatric patients)	84	180
Frequency of PD-L1 expression in tumor cells	64% of nonseminomas Some seminomas (no exact data)	76% of seminomas 89% of nonseminomas	Evaluated but no information	24.9% in total; 25.0% of seminomas; 24.8% of nonseminomas	No information (most likely expression of PD-L1 in TC was not observed)	0% (except ChC) with the use of dual PD-L1/OCT3/3 staining	12.2% (6 cases—3 ChC, 2 EC, 1 YS)	No information (most likely expression of PD-L1 in TC was not observed)	0% (except choriocarcinoma and focal staining in 3 EC)
Frequency of PD-L1 expression in TILs	73% in seminoma Rare in nonseminomas (no exact data)	No information	95.9% of seminomas; 91.0% of EC; 60% of YST; 54.5% of ChC; 35.7% of teratomas	85.5% in total; 87.2% of seminomas; 79.5% of nonseminomas	No exact information	Dependent on the pattern of staining (scattered/rare; intraseptal/stromal; extensive intratumoral)	8.16%	PD-L1 expression was described in “tumor parenchyma”	94.4% in total 92.8% of seminomas 96.4% of nonseminomas
Other immune markers assessed	No	PD-1—negative in tumor cells in all cases	PD-1—mainly low-expression in TILs in 87.7% of seminomatous tumors; 42.9% of EC; 38.8% teratomas; 26.9% of YST; 0% ChC	CTLA4—expressed in immune cells in 96.3% of GCTs and in tumor cells in 89.7% of cases; CD20 and CD3—lower levels associated with higher stage	PD-1, CD3, CD4, CD8, CD25, and FOXP3 Gene expression profiling with the NanoString pan-cancer immunology panel	PD-1, FOXP3, CD68, CD163	CD3, CD8, FOXP3	PD-1—expressed on TILs	VISTA—expressed in immune cells in 98.8% of GCTs
Other findings/Comments	No assessment of prognostic significance	High expression of PD-L1 in tumor cells is associated with worse PFS and OS	Patients with low PD-L1 in tumor cells and high PD-L1 in TILs have the best prognosis.	Absence of PD-L1 positive IC was associated with worse RFS. PD-L1 expression in TC had no prognostic impact.	Seminomas vs nonseminomas: higher levels of PD-L1+ IC. Advanced stage associated with more Tregs, decreased NK cell signature, increased neutrophil and macrophage signature	Higher expression of PD-L1+ TAMs in seminomas compared to nonseminomas. Activated TILs (FOXP3+) and TAMs are more abundant in nonmetastatic seminomas	Pediatric GCTs may be less immunogenic. A high CD3+ T-cell infiltration is associated with better outcome.	No association between PD-L1 or PD-1 expression and clinicopathological variables	Low expression of VISTA combined with high levels of PLR characterize stage II/III patients with high risk of relapse/progression Low PD-L1 expression may indicate risk of events in seminoma

## Data Availability

Additional information is available from corresponding author upon reasonable request

## References

[1] Dieckmann K.P., Pichlmeier U. (2004). Clinical epidemiology of testicular germ cell tumors. World J. Urol..

[2] Honecker F., Aparicio J., Berney D., Beyer J., Bokemeyer C., Cathomas R., Clarke N., Cohn-Cedermark G., Daugaard G., Dieckmann K.-P. (2018). ESMO Consensus Conference on testicular germ cell cancer: Diagnosis, treatment and follow-up. Ann. Oncol..

[3] Boormans J.L., Mayor de Castro J., Marconi L., Yuan Y., Laguna Pes M.P., Bokemeyer C., Nicolai N., Algaba F., Oldenburg J., Albers P. (2018). Testicular Tumour Size and Rete Testis Invasion as Prognostic Factors for the Risk of Relapse of Clinical Stage I Seminoma Testis Patients Under Surveillance: A Systematic Review by the Testicular Cancer Guidelines Panel. Eur. Urol..

[4] International Germ Cell Cancer Collaborative Group (1997). International Germ Cell Consensus Classification: A prognostic factor-based staging system for metastatic germ cell cancers. J. Clin. Oncol..

[5] Grivennikov S.I., Greten F.R., Karin M. (2010). Immunity, Inflammation, and Cancer. Cell.

[6] Ohno Y. (2019). Role of systemic inflammatory response markers in urological malignancy. Int. J. Urol..

[7] Zitvogel L., Kroemer G. (2012). Targeting PD-1/PD-L1 interactions for cancer immunotherapy. Oncoimmunology.

[8] Xu W., Hiếu T., Malarkannan S., Wang L. (2018). The structure, expression, and multifaceted role of immune-checkpoint protein VISTA as a critical regulator of anti-tumor immunity, autoimmunity, and inflammation. Cell. Mol. Immunol..

[9] Brockhoff G., Seitz S., Weber F., Zeman F., Klinkhammer-Schalke M., Ortmann O., Wege A.K. (2018). The presence of PD-1 positive tumor infiltrating lymphocytes in triple negative breast cancers is associated with a favorable outcome of disease. Oncotarget.

[10] Kim H.R., Ha S.-J., Hong M.H., Heo S.J., Koh Y.W., Choi E.C., Kim E.K., Pyo K.H., Jung I., Seo D. (2016). PD-L1 expression on immune cells, but not on tumor cells, is a favorable prognostic factor for head and neck cancer patients. Sci. Rep..

[11] Loeser H., Kraemer M., Gebauer F., Bruns C., Schröder W., Zander T., Persa O.-D., Alakus H., Hoelscher A., Buettner R. (2019). The expression of the immune checkpoint regulator VISTA correlates with improved overall survival in pT1/2 tumor stages in esophageal adenocarcinoma. Oncoimmunology.

[12] Sun C., Zhang L., Zhang W., Liu Y., Chen B., Zhao S., Li W., Wang L., Ye L., Jia K. (2020). Expression of PD-1 and PD-L1 on Tumor-Infiltrating Lymphocytes Predicts Prognosis in Patients with Small-Cell Lung Cancer. OncoTargets Ther..

[13] Zong L., Zhou Y., Zhang M., Chen J., Xiang Y. (2020). VISTA expression is associated with a favorable prognosis in patients with high-grade serous ovarian cancer. Cancer Immunol. Immunother..

[14] Lines J.L., Sempere L.F., Broughton T., Wang L., Noelle R. (2014). VISTA Is a Novel Broad-Spectrum Negative Checkpoint Regulator for Cancer Immunotherapy. Cancer Immunol. Res..

[15] ElTanbouly M.A., Croteau W., Noelle R.J., Lines J.L. (2019). VISTA: A novel immunotherapy target for normalizing innate and adaptive immunity. Semin. Immunol..

[16] ElTanbouly M.A., Zhao Y., Nowak E., Li J., Schaafsma E., Le Mercier I., Ceeraz S., Lines J.L., Peng C., Carriere C. (2020). VISTA is a checkpoint regulator for naïve T cell quiescence and peripheral tolerance. Science.

[17] Fankhauser C.D., Curioni-Fontecedro A., Allmann V., Beyer J., Tischler V., Sulser T., Moch H., Bode P.K. (2015). Frequent PD-L1 expression in testicular germ cell tumors. Br. J. Cancer.

[18] Cierna Z., Mego M., Miskovska V., Machalekova K., Chovanec M., Svetlovska D., Hainova K., Rejlekova K., Macak D., Spanik S. (2016). Prognostic value of programmed-death-1 receptor (PD-1) and its ligand 1 (PD-L1) in testicular germ cell tumors. Ann. Oncol..

[19] Lobo J., Rodrigues Â., Guimarães R., Cantante M., Lopes P., Maurício J., Oliveira J., Jerónimo C., Henrique R. (2019). Detailed Characterization of Immune Cell Infiltrate and Expression of Immune Checkpoint Molecules PD-L1/CTLA-4 and MMR Proteins in Testicular Germ Cell Tumors Disclose Novel Disease Biomarkers. Cancers.

[20] Hinsch A., Blessin N., Simon R., Kluth M., Fischer K., Hube-Magg C., Li W., Makrypidi-Fraune G., Wellge B., Mandelkow T. (2019). Expression of the immune checkpoint receptor TIGIT in seminoma. Oncol. Lett..

[21] Siska P.J., Johnpulle R.A.N., Zhou A., Bordeaux J., Kim J.Y., Dabbas B., Dakappagari N., Rathmell J.C., Rathmell W.K., Morgans A.K. (2017). Deep exploration of the immune infiltrate and outcome prediction in testicular cancer by quantitative multiplexed immunohistochemistry and gene expression profiling. Oncoimmunology.

[22] Gabay C., Kushner I. (1999). Acute-Phase Proteins and Other Systemic Responses to Inflammation. N. Engl. J. Med..

[23] Li B., Zhou P., Liu Y., Wei H., Yang X., Chen T., Xiao J. (2018). Platelet-to-lymphocyte ratio in advanced Cancer: Review and meta-analysis. Clin. Chim. Acta.

[24] Templeton A.J., Ace O., McNamara M.G., Al-Mubarak M., Vera-Badillo F.E., Hermanns T., Šeruga B., Ocaña A., Tannock I.F., Amir E. (2014). Prognostic role of platelet to lymphocyte ratio in solid tumors: A systematic review and meta-analysis. Cancer Epidemiol. Biomark. Prev..

[25] Brighi N., Farolfi A., Conteduca V., Gurioli G., Gargiulo S., Gallà V., Schepisi G., Lolli C., Casadei C., De Giorgi U. (2019). The Interplay between Inflammation, Anti-Angiogenic Agents, and Immune Checkpoint Inhibitors: Perspectives for Renal Cell Cancer Treatment. Cancers.

[26] Moschetta M., Uccello M., Kasenda B., Mak G., McClelland A., Boussios S., Forster M., Arkenau H.-T. (2017). Dynamics of Neutrophils-to-Lymphocyte Ratio Predict Outcomes of PD-1/PD-L1 Blockade. BioMed Res. Int..

[27] Lee D.Y., Im E., Yoon D., Lee Y.-S., Kim G.-S., Kim D., Kim S.-H. (2020). Pivotal role of PD-1/PD-L1 immune checkpoints in immune escape and cancer progression: Their interplay with platelets and FOXP3+Tregs related molecules, clinical implications and combinational potential with phytochemicals. Semin. Cancer Biol..

[28] Lalani A.-K.A., Xie W., Martini D.J., Steinharter J.A., Norton C.K., Krajewski K.M., Duquette A., Bossé D., Bellmunt J., Van Allen E.M. (2018). Change in neutrophil-to-lymphocyte ratio (NLR) in response to immune checkpoint blockade for metastatic renal cell carcinoma. J. Immunother. Cancer.

[29] Chovanec M., Cierna Z., Miskovska V., Machalekova K., Kalavska K., Rejlekova K., Svetlovska D., Macak D., Spanik S., Kajo K. (2018). Systemic immune-inflammation index in germ-cell tumours. Br. J. Cancer.

[30] Paner G.P., Stadler W.M., Hansel D.E., Montironi R., Lin D.W., Amin M.B. (2018). Updates in the Eighth Edition of the Tumor-Node-Metastasis Staging Classification for Urologic Cancers. Eur. Urol..

[31] R Core Team (2020). R: A Language and Environment for Statistical Computing.

[32] Wickham H. (2016). ggplot2: Elegant Graphics for Data Analysis.

[33] Kassambara A., Kosinski M., Biecek P. (2020). Survminer: Drawing Survival Curves Using “ggplot2”. https://cran.r-project.org/web/packages/survminer/.

[34] Xiao N. (2018). ggplot2Scientific Journal and Sci-Fi ThemedColor Palettes for "ggplot2. R Package Version 2.7.

[35] Qu N., Ogawa Y., Kuramasu M., Nagahori K., Sakabe K., Itoh M. (2020). Immunological microenvironment in the testis. Reprod. Med. Biol..

[36] Zhao S., Zhu W., Xue S., Han D. (2014). Testicular defense systems: Immune privilege and innate immunity. Cell. Mol. Immunol..

[37] Brunet-Possenti F., Opsomer M.A., Gomez L., Ouzaid I., Descamps V. (2016). Immune checkpoint inhibitors-related orchitis. Ann. Oncol..

[38] Kalavska K., Schmidtova S., Chovanec M., Mego M. (2020). Immunotherapy in Testicular Germ Cell Tumors. Front. Oncol..

[39] Dorantes-Heredia R., Motola-Kuba D., Murphy-Sanchez C., Izquierdo-Tolosa C.D., Ruiz-Morales J.M. (2019). Spontaneous regression as a ‘burned-out’ non-seminomatous testicular germ cell tumor: A case report and literature review. J. Surg. Case Rep..

[40] Marshall A.H.., Dayan A. (1964). An immune reaction in man against seminomas, dysgerminomas, pinealomas, and the mediastinal tumours of similar histological appearance?. Lancet.

[41] Hadrup S.R., Brændstrup O., Jacobsen G.K., Mortensen S., Pedersen L.Ø., Seremet T., Andersen M.H., Becker J.C., Straten P. (2006). Tumor infiltrating lymphocytes in seminoma lesions comprise clonally expanded cytotoxic T cells. Int. J. Cancer.

[42] Pearce H., Hutton P., Chaudhri S., Porfiri E., Patel P., Viney R., Moss P. (2017). Spontaneous CD4+ and CD8+ T-cell responses directed against cancer testis antigens are present in the peripheral blood of testicular cancer patients. Eur. J. Immunol..

[43] Cheng X., Dai H., Wan N., Moore Y., Vankayalapati R., Dai Z. (2009). Interaction of Programmed Death-1 and Programmed Death-1 Ligand-1 Contributes to Testicular Immune Privilege. Transplantation.

[44] Chovanec M., Cierna Z., Miskovska V., Machalekova K., Svetlovska D., Kalavska K., Rejlekova K., Spanik S., Kajo K., Babal P. (2017). Prognostic role of programmed-death ligand 1 (PD-L1) expressing tumor infiltrating lymphocytes in testicular germ cell tumors. Oncotarget.

[45] Sadigh S., Farahani S.J., Shah A., Vaughn D., Lal P. (2020). Differences in PD-L1–Expressing Macrophages and Immune Microenvironment in Testicular Germ Cell Tumors. Am. J. Clin. Pathol..

[46] Villarroel-Espindola F., Yu X., Datar I., Mani N., Sanmamed M., Velcheti V., Syrigos K., Toki M., Zhao H., Chen L. (2018). Spatially Resolved and Quantitative Analysis of VISTA/PD-1H as a Novel Immunotherapy Target in Human Non–Small Cell Lung Cancer. Clin. Cancer Res..

[47] Cao X., Ren X., Zhou Y., Mao F., Lin Y., Wu H., Sun Q. (2021). VISTA Expression on Immune Cells Correlates With Favorable Prognosis in Patients With Triple-Negative Breast Cancer. Front. Oncol..

[48] Liu C.-Q., Xu J., Zhou Z.-G., Jin L.-L., Yu X.-J., Xiao G., Lin J., Zhuang S.-M., Zhang Y.-J., Zheng L. (2018). Expression patterns of programmed death ligand 1 correlate with different microenvironments and patient prognosis in hepatocellular carcinoma. Br. J. Cancer.

[49] Pollari M., Brück O., Pellinen T., Vähämurto P., Karjalainen-Lindsberg M.-L., Mannisto S., Kallioniemi O., Kellokumpu-Lehtinen P.-L., Mustjoki S., Leivonen S.-K. (2018). PD-L1 + tumor-associated macrophages and PD-1 + tumor-infiltrating lymphocytes predict survival in primary testicular lymphoma. Haematologica.

[50] Boldrini R., De Pasquale M.D., Melaiu O., Chierici M., Jurman G., Benedetti M.C., Salfi N.C., Castellano A., Collini P., Furlanello C. (2019). Tumor-infiltrating T cells and PD-L1 expression in childhood malignant extracranial germ-cell tumors. Oncoimmunology.

[51] Jennewein L., Bartsch G., Gust K., Kvasnicka H., Haferkamp A., Blaheta R., Mittelbronn M., Harter P., Mani J. (2018). Increased tumor vascularization is associated with the amount of immune competent PD-1 positive cells in testicular germ cell tumors. Oncol. Lett..

[52] Liu J., Yuan Y., Chen W., Putra J., Suriawinata A.A., Schenk A.D., Miller H.E., Guleria I., Barth R.J., Huang Y.H. (2015). Immune-checkpoint proteins VISTA and PD-1 nonredundantly regulate murine T-cell responses. Proc. Natl. Acad. Sci. USA.

[53] Zong L., Zhang M., Wang W., Wan X., Yang J., Xiang Y. (2019). PD -L1, B7-H3 and VISTA are highly expressed in gestational trophoblastic neoplasia. Histopathology.

[54] Necchi A., Giannatempo P., Raggi D., Mariani L., Colecchia M., Farè E., Monopoli F., Calareso G., Ali S.M., Ross J.S. (2019). An Open-label Randomized Phase 2 study of Durvalumab Alone or in Combination with Tremelimumab in Patients with Advanced Germ Cell Tumors (APACHE): Results from the First Planned Interim Analysis. Eur. Urol..

[55] Adra N., Einhorn L.H., Althouse S.K., Ammakkanavar N.R., Musapatika D., Albany C., Vaughn D., Hanna N.H. (2018). Phase II trial of pembrolizumab in patients with platinum refractory germ-cell tumors: A Hoosier Cancer Research Network Study GU14-206. Ann. Oncol..

[56] Zschäbitz S., Lasitschka F., Hadaschik B., Hofheinz R.-D., Jentsch-Ullrich K., Grüner M., Jäger D., Grüllich C. (2017). Response to anti-programmed cell death protein-1 antibodies in men treated for platinum refractory germ cell cancer relapsed after high-dose chemotherapy and stem cell transplantation. Eur. J. Cancer.

[57] Tagliamento M., Bironzo P., Novello S. (2019). New emerging targets in cancer immunotherapy: The role of VISTA. ESMO Open.

[58] Tashima Y., Kuwata T., Yoneda K., Hirai A., Mori M., Kanayama M., Imanishi N., Kuroda K., Ichiki Y., Tanaka F. (2020). Prognostic impact of PD-L1 expression in correlation with neutrophil-to-lymphocyte ratio in squamous cell carcinoma of the lung. Sci. Rep..

[59] Hasegawa T., Yanagitani N., Utsumi H., Wakui H., Sakamoto H., Tozuka T., Yoshida H., Amino Y., Uematsu S., Yoshizawa T. (2019). Association of High Neutrophil-to-Lymphocyte Ratio With Poor Outcomes of Pembrolizumab Therapy in High-PD-L1-expressing Non-small Cell Lung Cancer. Anticancer Res..

[60] Yuksel O.H., Verit A., Sahin A., Urkmez A., Uruc F. (2016). White blood cell counts and neutrophil to lymphocyte ratio in the diagnosis of testicular cancer: A simple secondary serum tumor marker. Int. Braz. J. Urol..

[61] Gokcen K., Dundar G., Gulbahar H., Gokce G., Gultekin E.Y. (2018). Can routine peripheral blood counts like neutrophil-to-lymphocyte ratio be beneficial in prediagnosis of testicular cancer and its stages?. J. Res. Med. Sci..

[62] Herraiz-Raya L., Moreillo-Vicente L., Martínez-Ruiz J., Agustí-Martínez A., Fernández-Anguita P.J., Esper-Rueda J.A., Salce-Marte L., Armas-Álvarez A., Díaz de Mera-Sánchez Migallón I., Martínez-Alfaro C. (2019). Leukocyte and platelet counts as prognostic values of testicular germ cell tumors. Actas Urológicas Españolas.

[63] Tan Y.G., Sia J., Huang H.H., Lau W.K.O. (2019). Neutrophil-to-lymphocyte ratio independently predicts advanced pathological staging and poorer survival outcomes in testicular cancer. Investig. Clin. Urol..

[64] Fankhauser C.D., Sander S., Roth L., Gross O., Eberli D., Sulser T., Seifert B., Beyer J., Hermanns T. (2018). Systemic inflammatory markers have independent prognostic value in patients with metastatic testicular germ cell tumours undergoing first-line chemotherapy. Br. J. Cancer.

[65] Maruyama Y., Sadahira T., Araki M., Mitsui Y., Wada K., Edamura K., Kobayashi Y., Watanabe M., Watanabe T., Nasu Y. (2019). Comparison of the predictive value among inflammation-based scoring systems for bleomycin pulmonary toxicity in patients with germ cell tumors. Int. J. Urol..

[66] Nieswandt B., Hafner M., Echtenacher B., Männel D.N. (1999). Lysis of tumor cells by natural killer cells in mice is impeded by platelets. Cancer Res..

[67] Labelle M., Begum S., Hynes R.O. (2011). Direct Signaling between Platelets and Cancer Cells Induces an Epithelial-Mesenchymal-Like Transition and Promotes Metastasis. Cancer Cell.

[68] Badia R.R., Woldu S., Patel H.D., Singla N., Srivastava A., Cheaib J.G., Pierorazio P.M., Bagrodia A. (2021). Clinical utility of the AJCC 8th edition pT1 subclassification and impact on practice patterns in stage I seminoma. Urol. Oncol. Semin. Orig. Investig..

